# Supplementary material for: Middle Ordovician astrochronology decouples asteroid breakup from glacially-induced biotic radiations
Source: Nat Commun. 2021 Nov 5;12:6430. doi: 10.1038/s41467-021-26396-4 (PMC8571325; doi:10.1038/s41467-021-26396-4)
Supplement: Supplementary file 1 — Supplementary Information [file 41467_2021_26396_MOESM1_ESM.pdf]

Middle Ordovician astrochronology decouples asteroid breakup from glacially-induced biotic radiations

By Rasmussen, JA, Thibault, N and Rasmussen, CMØ

Supplementary Information

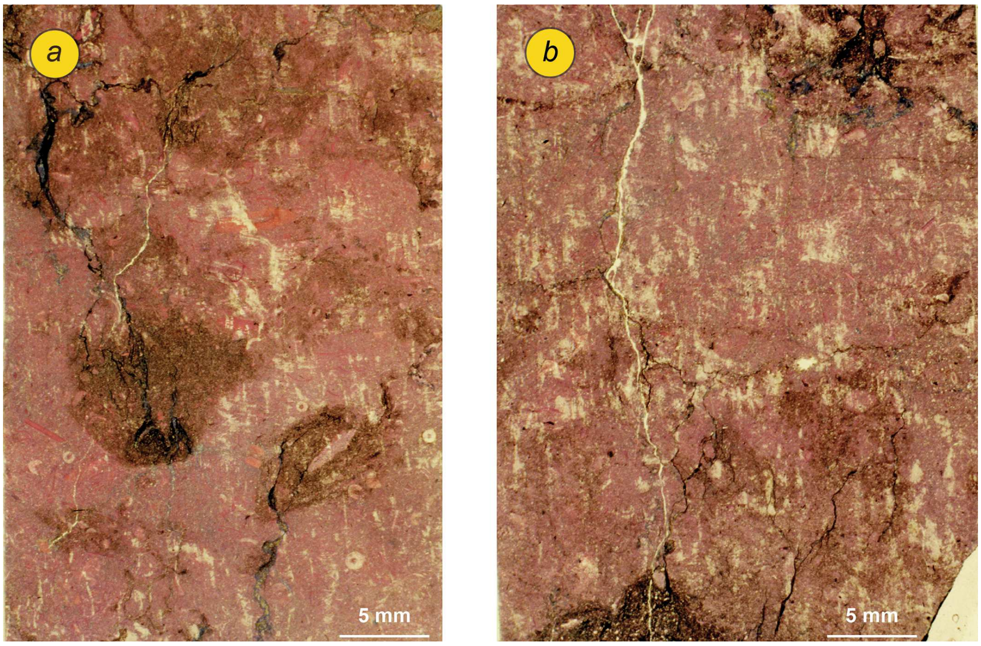

Supplementary Fig. 1: Various lithofacies. a Lithofacies f. Thin section from near the 32.5 m level dominated by pure limestone (wackestone; 85 % $CaCO_3$). All $CaCO_3$%-values shown in the Supplementary Figures 1–3 captions are based on Atomic absorption spectrophotometer analyses[1]. The vertical thin section is stained with Alizarin Red, and red coloured areas show the occurrence of calcium-carbonate, while brown areas represent seams made of siliciclastic clay or silt. The Lithofacies f is however dominating. Pressure dissolution seams are visible in the upper left-hand part of the photograph. Remark the common circular crinoid fragments. Clear blue colour indicates empty cracks and pores. **b** Lithofacies f. Thin section from the 27 m level dominated by pure limestone (mudstone). $CaCO_3$ content data are not available from this sample.

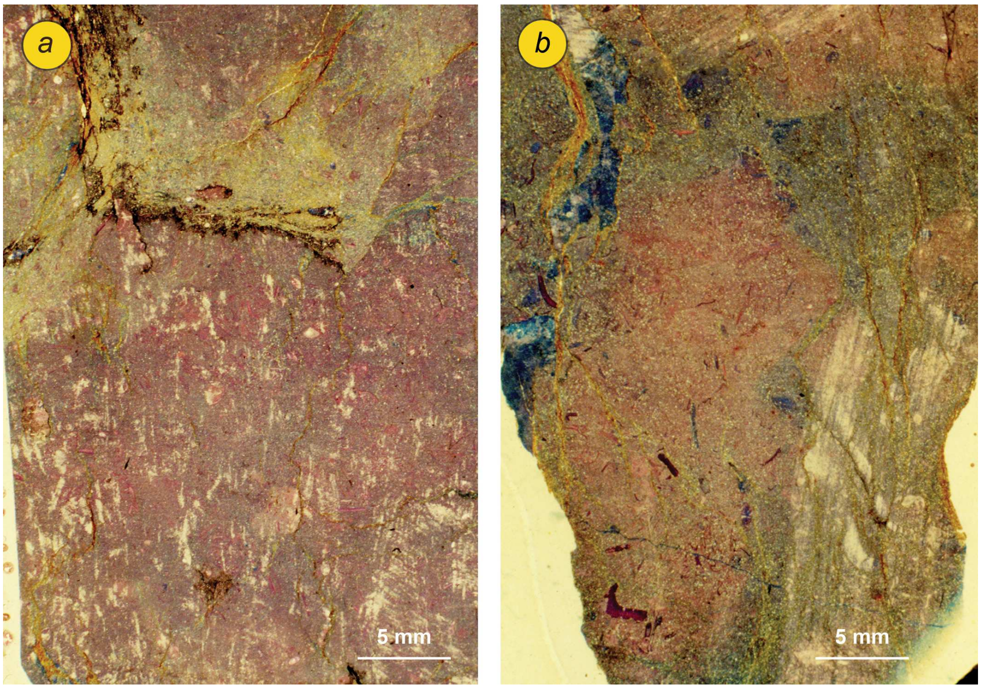

Supplementary Fig. 2: Various lithofacies. a Lithofacies e. Thin section from near the 18.4 m level dominated by relatively pure limestone (mud- and wackestone; 70 % $CaCO_3$) with thin clay seams. **b** Lithofacies d. Thin section from the 39.2 m level. The sample comprises thin-bedded, relatively pure limestone (mud- and wackestone; 60 % $CaCO_3$) dominated by lithofacies d. Shelly fossil fragments are common. An open crack is seen in the upper left-hand corner (filled with blue epoxy).

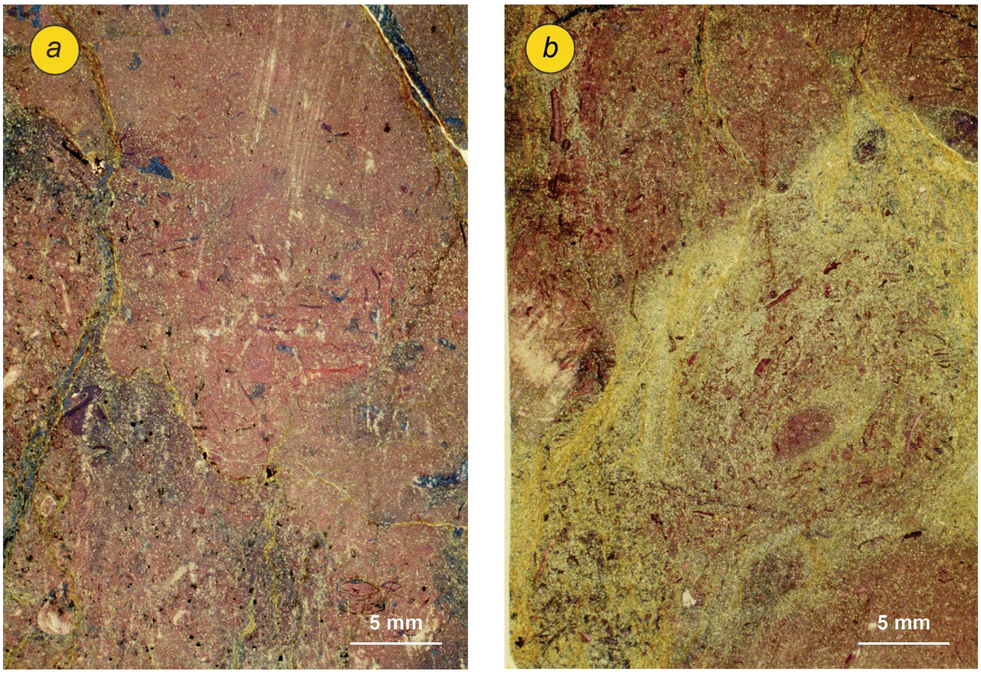

Supplementary Fig. 3: Various lithofacies. a Lithofacies d. Thin section from near the 40.50 m level dominated by thin-bedded, relatively pure limestone (wackestone) containing common fossil fragments (59 % $CaCO_3$). **b** Lithofacies c. Thin section from near the 37.6 m level. The sample consists of coarsely reticulate, impure limestone (mudstone) dominated by lithofacies c. Small shelly fossil fragments are common both in the carbonate-rich and clay-rich portions. $CaCO_3$ content data are not available from this sample.

Lithofacies	Description	Lithologic rank	Colour code in figures
a1	Cracks and eroded claystone	-1	
a2	Shale and siliciclastic claystone	-1	
a3	Dark, thin, siliciclastic siltstone seam	-1	
b	Marly limestone, finely reticulate	-0.5	
c	Marly limestone, coarsely reticulate	0	
d	Relatively pure limestone, thin-bedded	0.5	
e	Relatively pure limestone with few thin clay seams	1	
f	Pure massive limestone	1.5	

Supplementary Table 1: Description of the distinct lithofacies recognized in the field with their respective rank and color code used in supplementary figures.

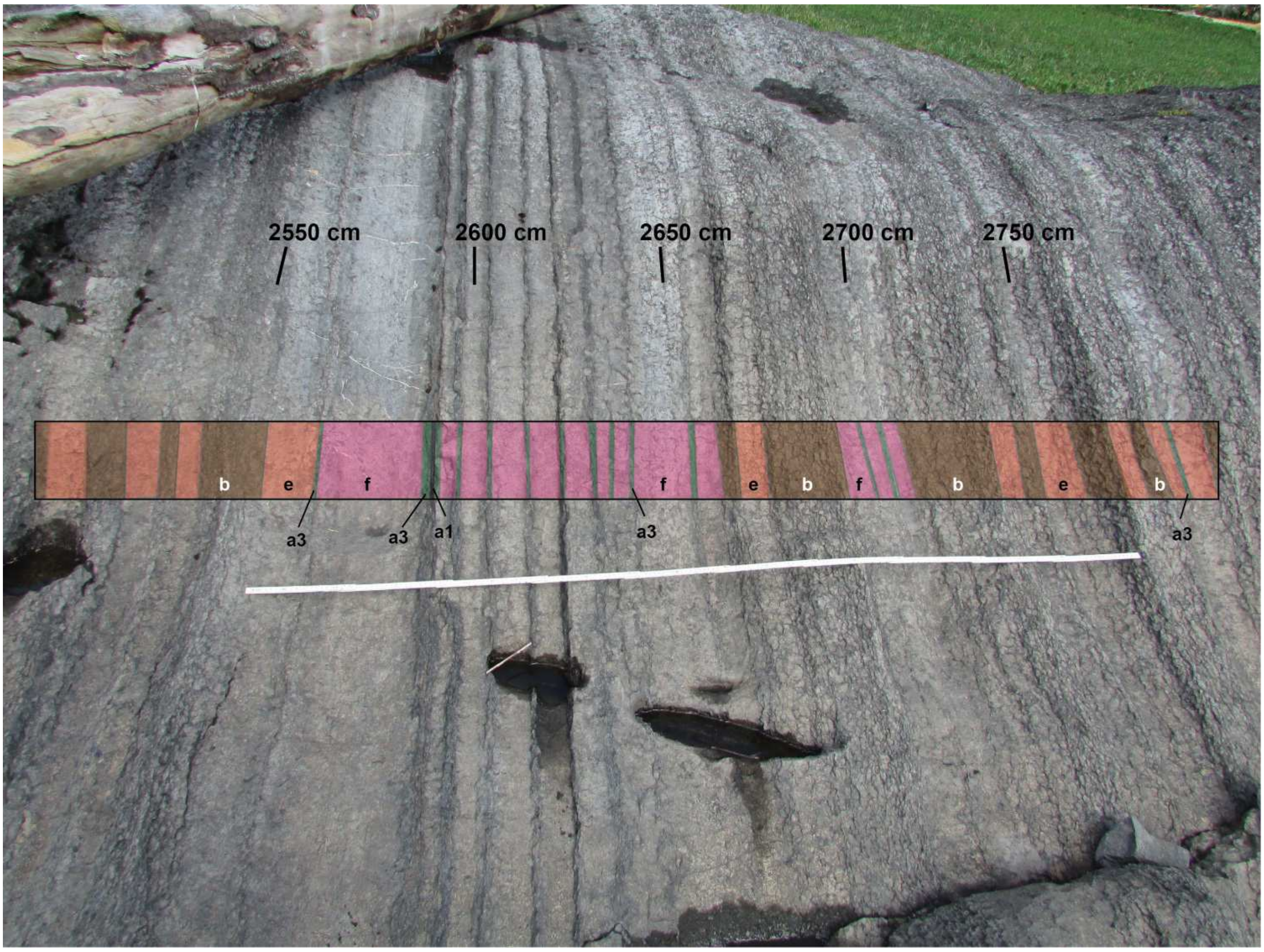

Supplementary Fig. 4: The upper part of the measured section (25.5 – 27.5 m) as in Figure 5 of the main text. The vertical bedding in this part of the Stein Formation demonstrates that light grey, rather pure limestone facies are interbedded with darker clayey and silty marls and limestones in a cyclic pattern. The letters and colours in the lower part of the picture depict the various lithofacies occurring in this part of the section (see explanation of the letters in Supplementary Table 1). The white ruler in the lowermost part of the photo is 2 m long. See Supplementary Fig. 1A–B for thin sections showing details of Lithofacies f.

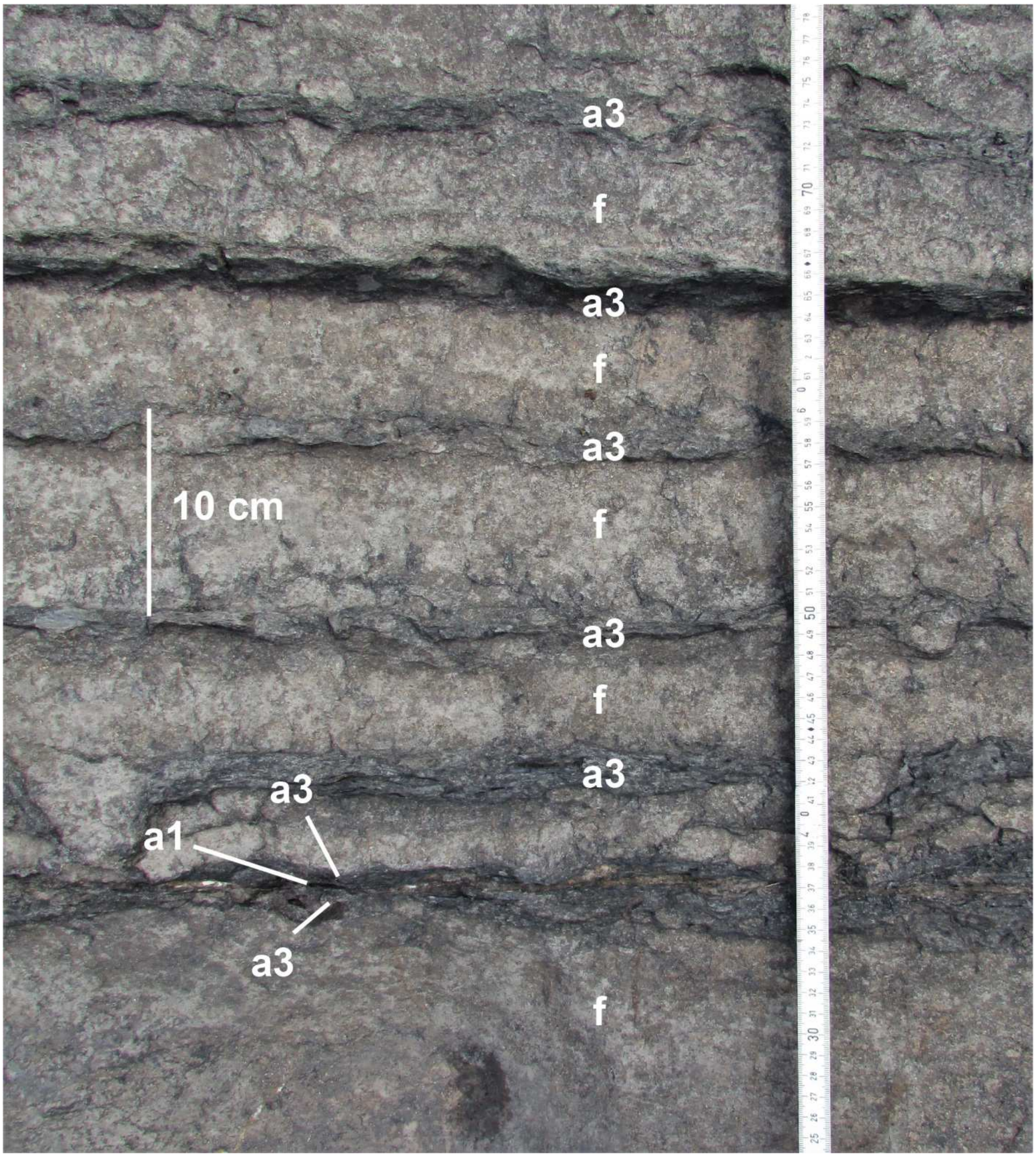

Supplementary Fig. 5: Close-up picture of the section near the 26 m level showing regular cycles between pure limestone beds (Lithofacies f) and dark, thin siliciclastic, silt- and claystone layers of Lithofacies a1–a3.

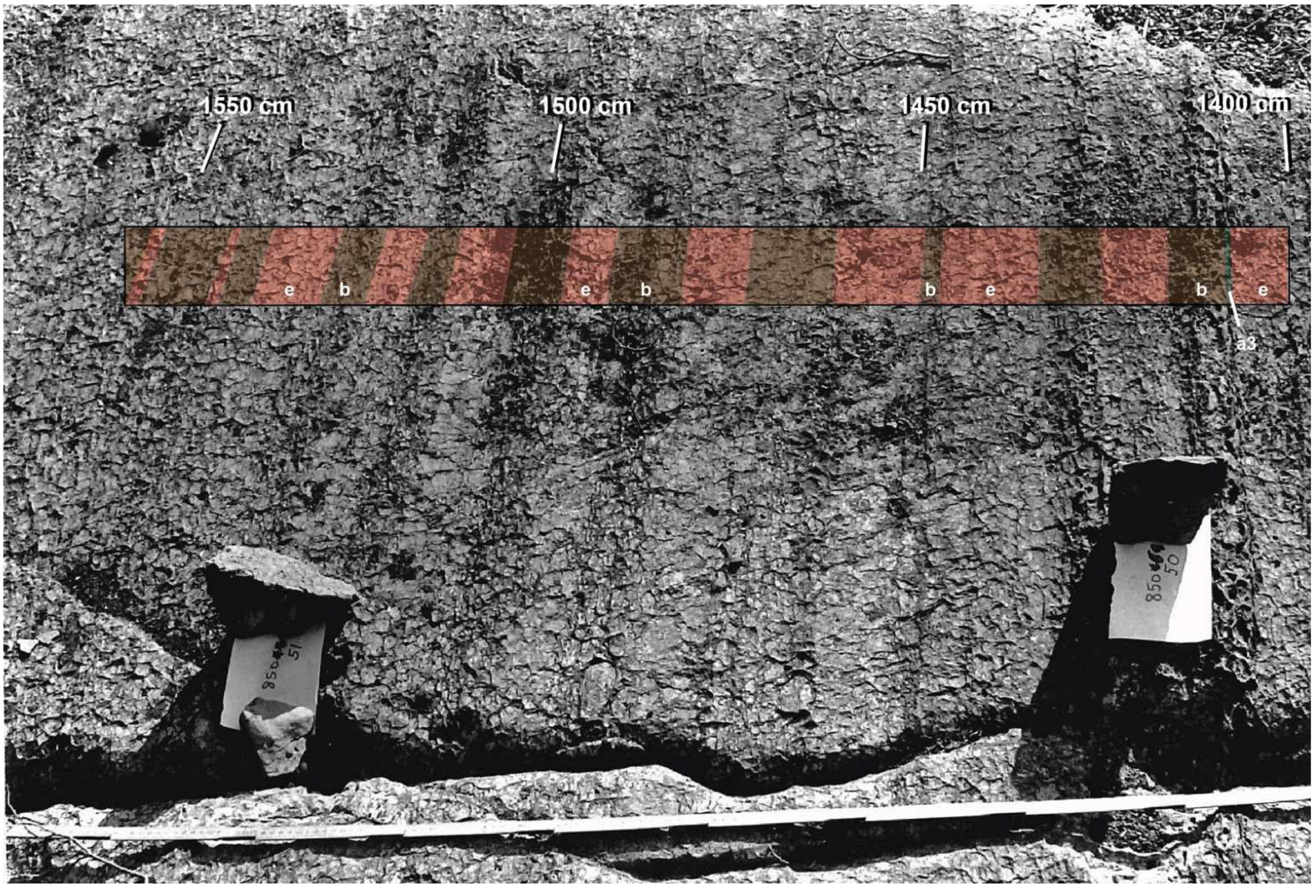

Supplementary Fig. 6: Vertical bedding in the middle part of the measured section (14.0 – 15.5 m) showing regular bedding alternating between relatively pure limestone with few, thin clay seams (Lithofacies a3) and finely reticulate, impure limestone (Lithofacies b). The letters and colours demonstrate the various lithofacies occurring in this part of the section (see explanation of the letters in Supplementary Table 1). See **Supplementary Fig. 2**A–B for thin sections showing details of Lithofacies e and d.

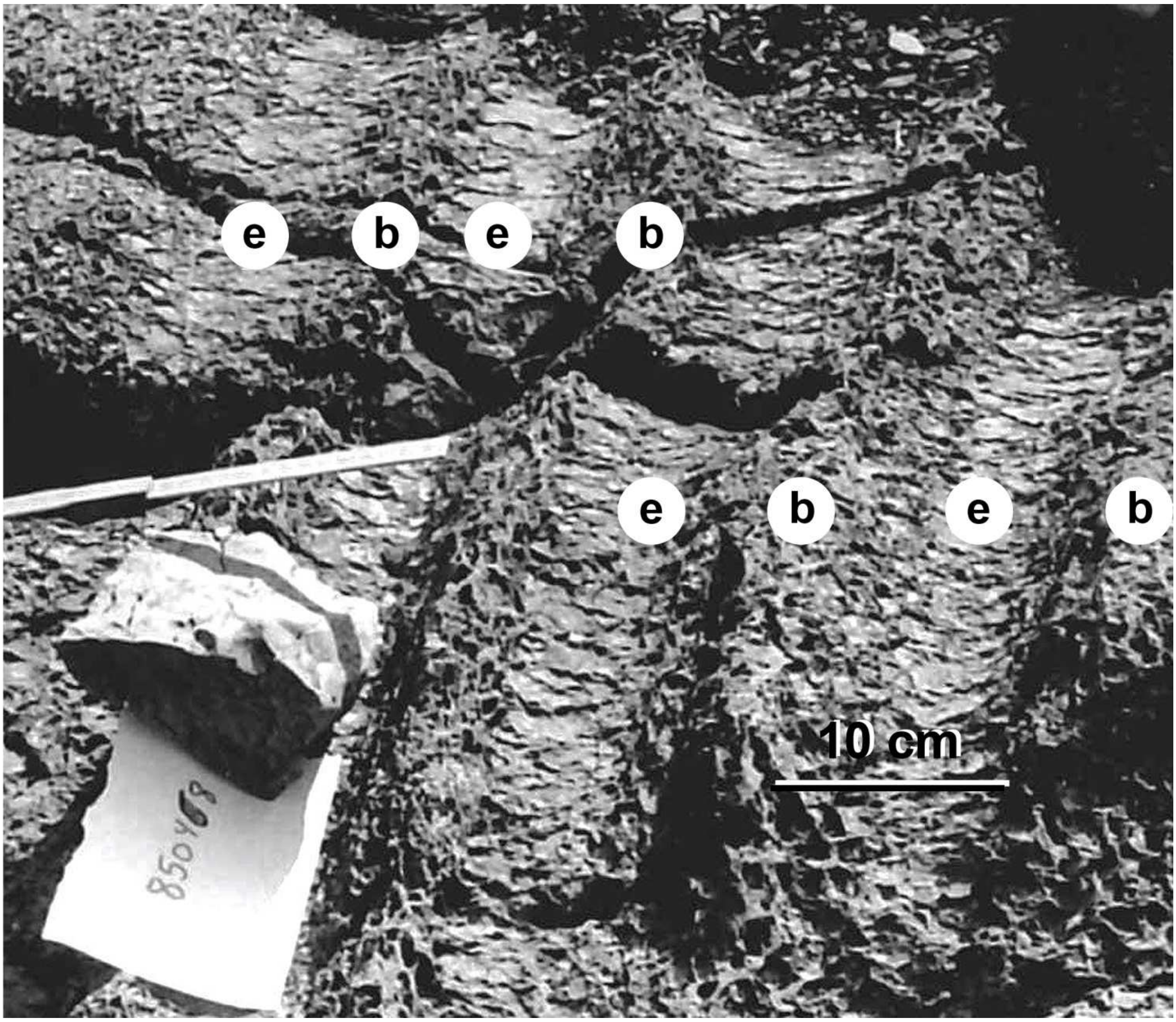

Supplementary Fig. 7: Close-up picture of the vertical section near the 12 m level showing regular cycles between relatively pure limestone beds with thin clay seams (Lithofacies e) and distinctly reticulate, impure limestone (Lithofacies b), the latter being most resistant to erosion.

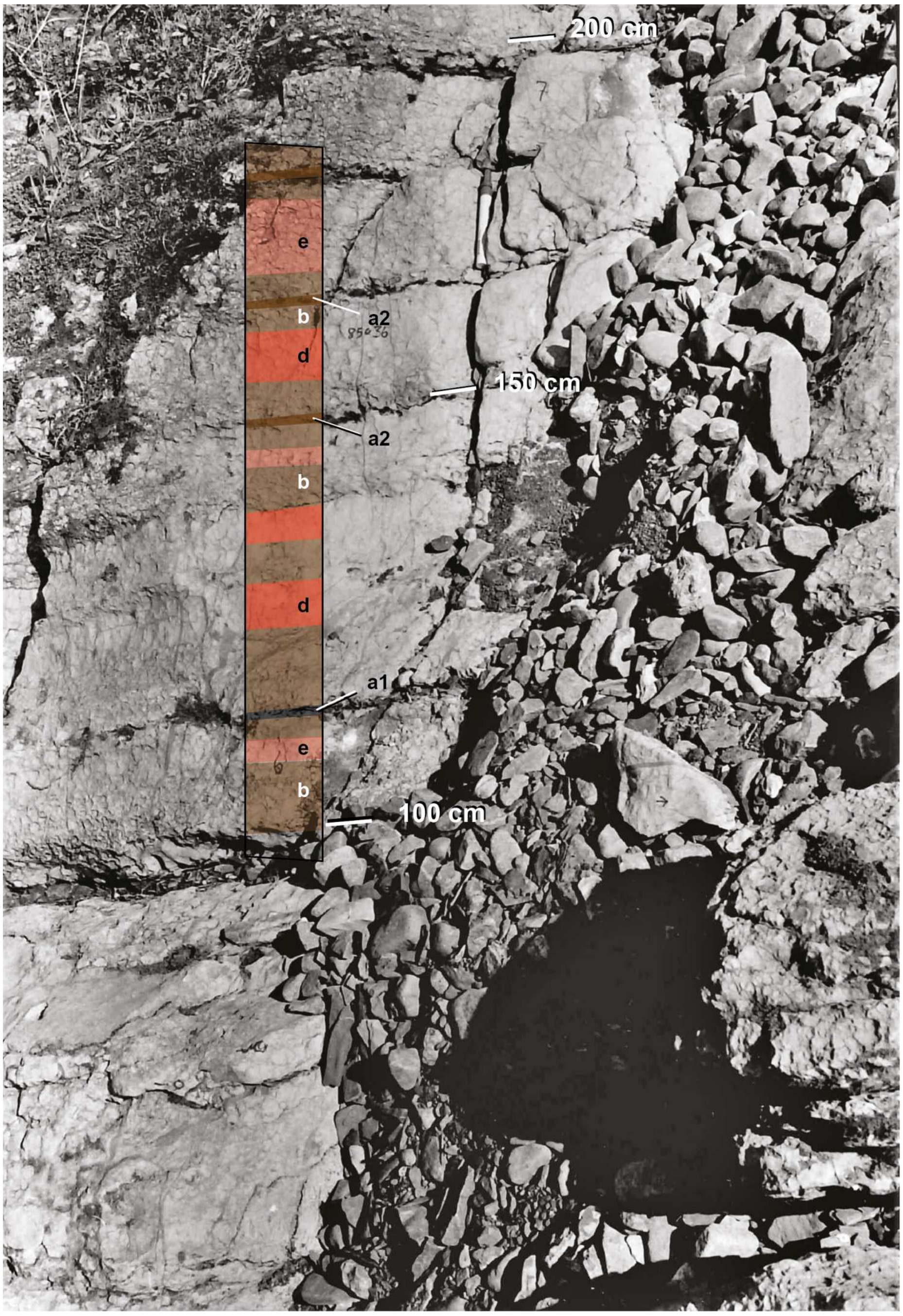

Supplementary Fig. 8: Photograph of the lowermost part of the analysed section (1.0–2.0 m) showing beds of near pure limestone (Lithofacies d and e) alternating with reticulate, impure limestone (Lithofacies b). Thin layers dominated by siliciclastic claystone (Lithofacies a2) occur in the middle portion of the impure limestone beds. The letters and colours demonstrate various lithofacies occurring in this part of the section (see explanation of the letters in Supplementary Table 1). See Supplementary Fig. 3A-B for thin sections showing details of Lithofacies d.

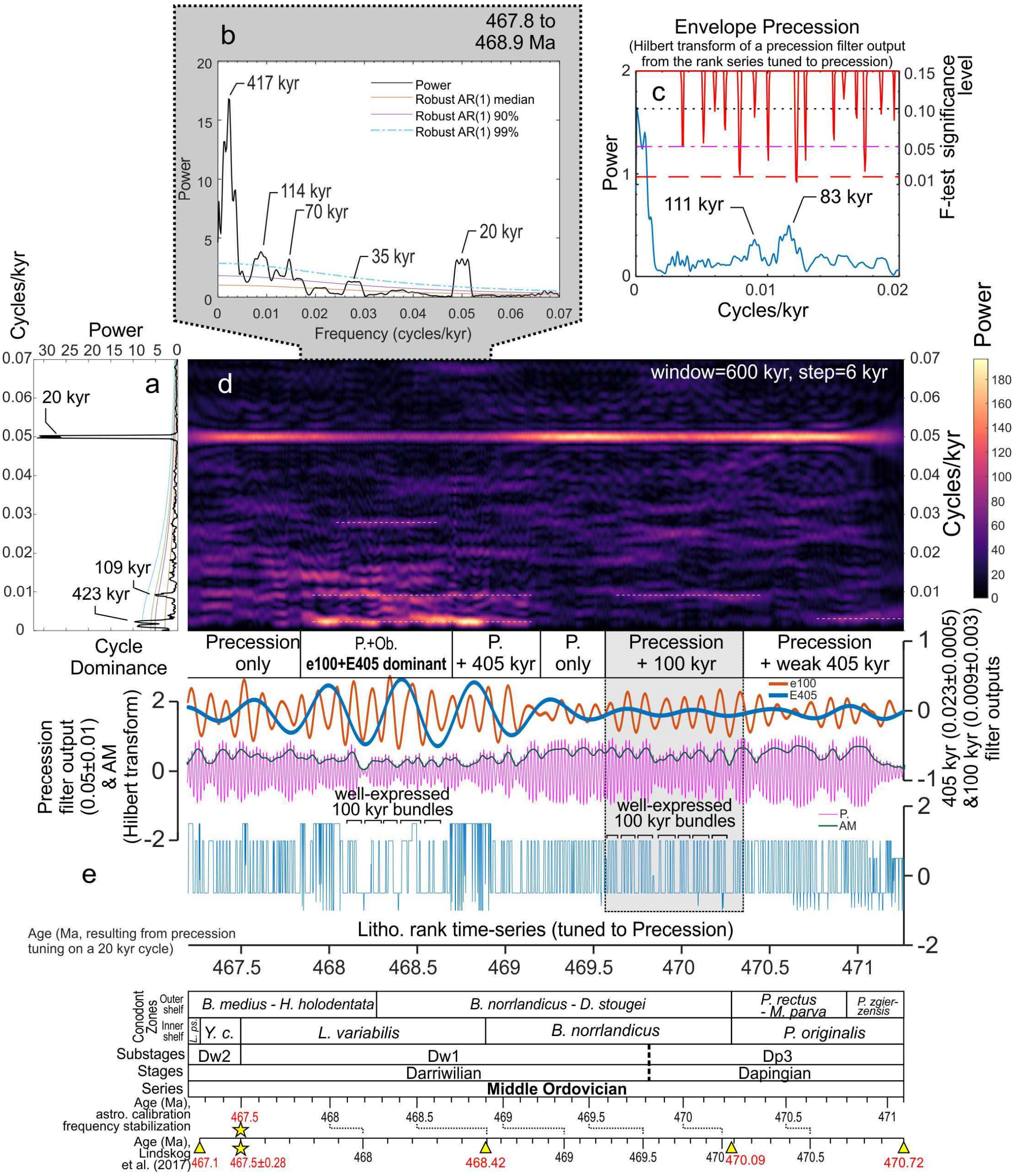

Supplementary Fig. 9: Results of a cyclostratigraphic analysis in the time domain after tuning to precession cycles. **a** 2 π MTM periodogram of the time-series tuned to 20 kyr precession cycles. **b** 2 π MTM periodogram of the selected interval at 467.8 to 468.9 Ma showing the expression of an obliquity component at 35 kyr. **c** 2 π MTM periodogram with F-test significance level of individual peaks for the amplitude modulations of the precession filter output (see panel E) showing the expression of the 100 kyr. **d** Evo-FFT of the tuned time-series highlighting the expression of the different orbital components along the time-series. **e** Tuned time-series with filter outputs and interpreted cycle dominance.

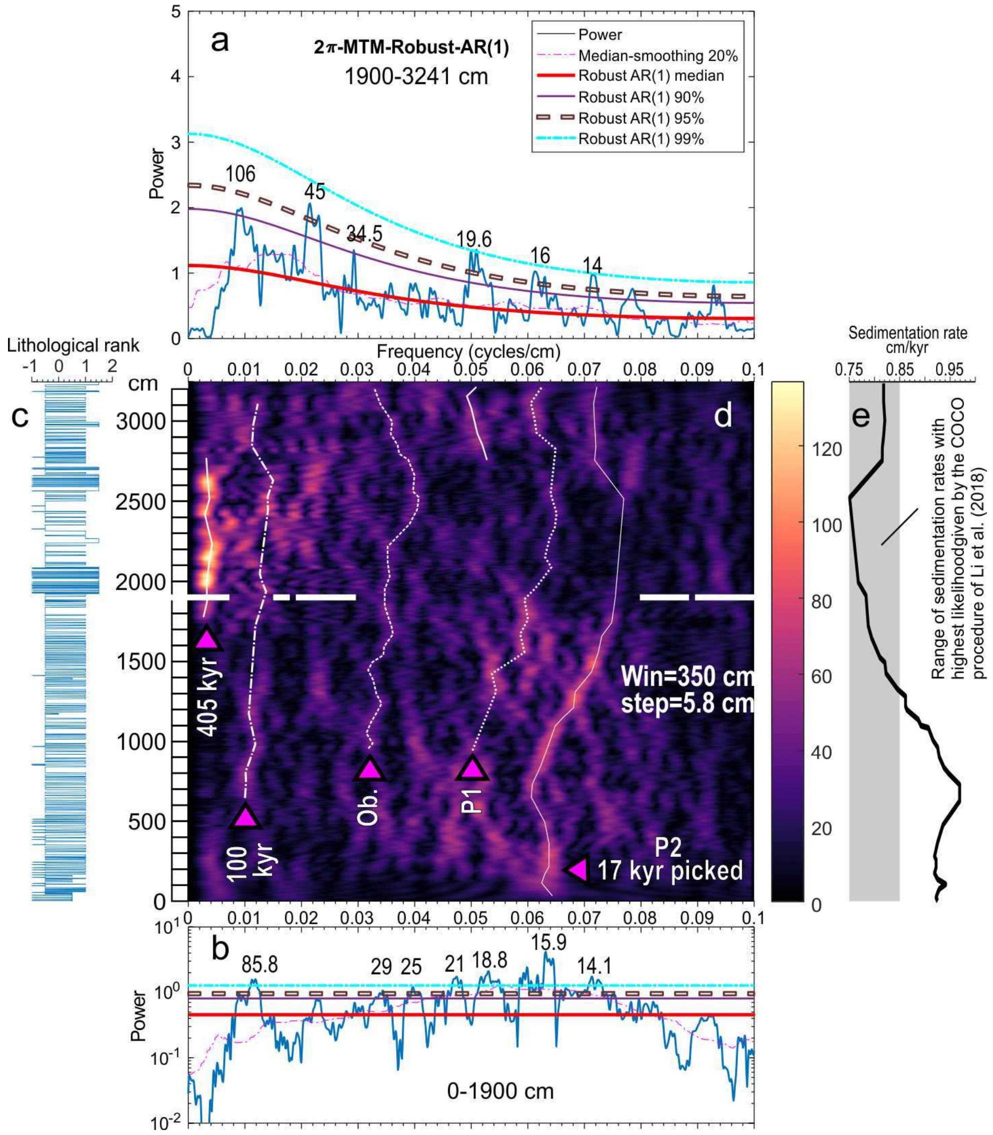

Supplementary Fig. 10: Frequency stabilization procedure applied for the retained astronomical calibration of the Steinsodden section. **a–b** 2 π MTM periodograms of the detrended time-series for the two distinct intervals 0–1900 cm and 1900–3241 cm. Detrending was performed using a highpass filter with a cut at 0.004 cycles/cm so as to remove the influence of long-term trends and of the 405 kyr and highlight better other orbital components. **c** Time-series in the depth domain. **d** Evo-FFT of the time-series in the depth domain showing various interpreted orbital components and how the highest significant frequency was interpreted as the 17 kyr component of the precession (P2) and was picked along the evo-FFT. **e** Variations in sedimentation rate resulting from the picking

procedure and used to establish an age-model for the tuning in Acycle. In grey is shown the optimal range of sedimentation rates derived from the COCO procedure[2] (see Supplementary Fig. 12).

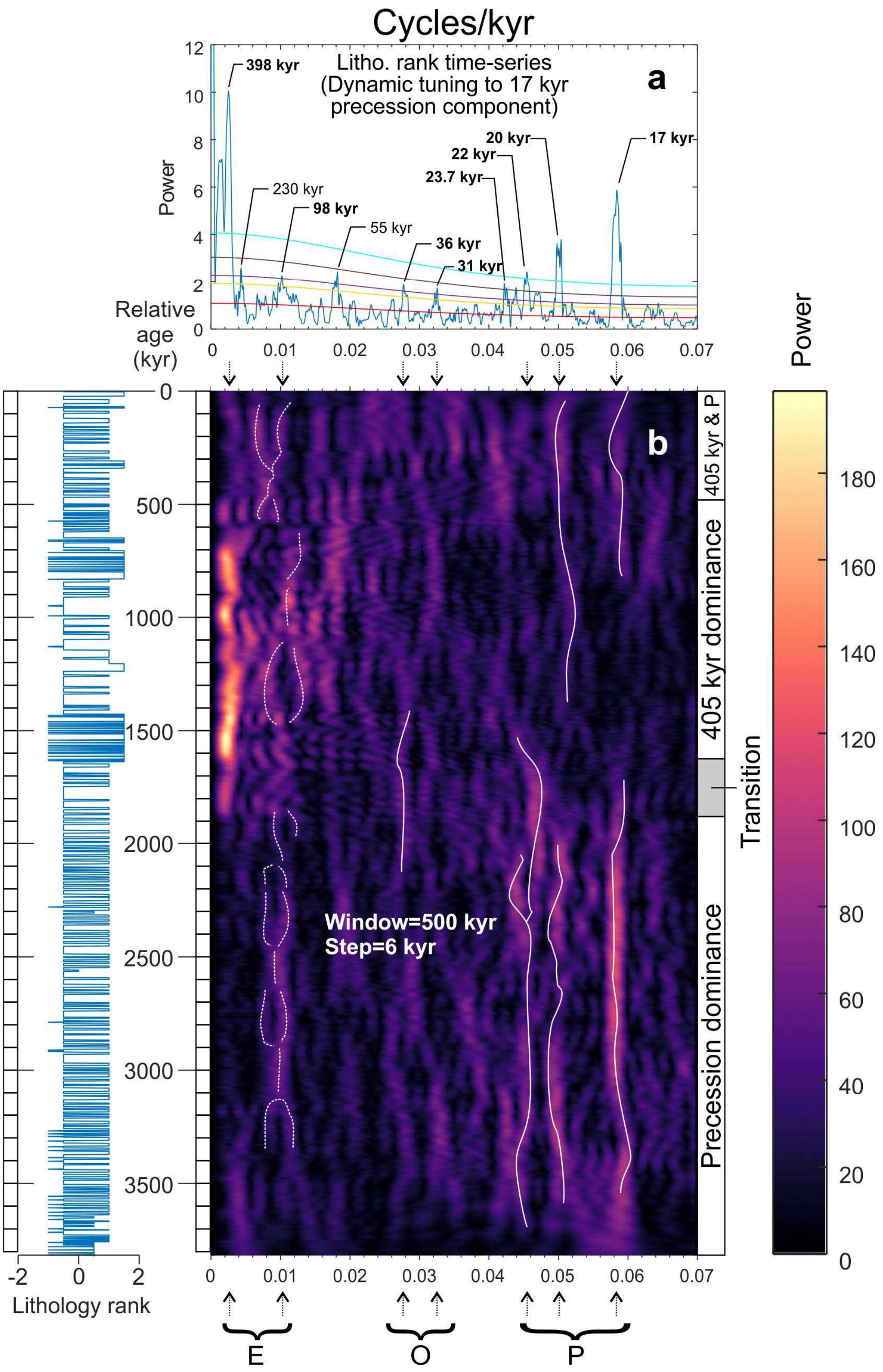

Supplementary Fig. 11: Cyclostratigraphic of the dynamically-tuned timeseries in the time domain.

a 2 π MTM periodogram and **b** evo-FFT of the time-series dynamically tuned by frequency stabilization of the 17 kyr precession component. Note that this procedure preserves several distinct

components in the precession frequency band. A possible 36 kyr obliquity component appears particularly well-expressed within the transition interval at 1600–2100 kyr in relative age. The short-eccentricity component shows a poorly significant peak at 98 kyr in the periodogram but beautiful amplitude modulations by the 405 kyr in the evo-FFT while the 405 kyr component is expressed at the base of the time-series below 3500 kyr and dominates in the upper half, except for the topmost 500 kyr where both 405 kyr and precession components appear to be expressed with equal power.

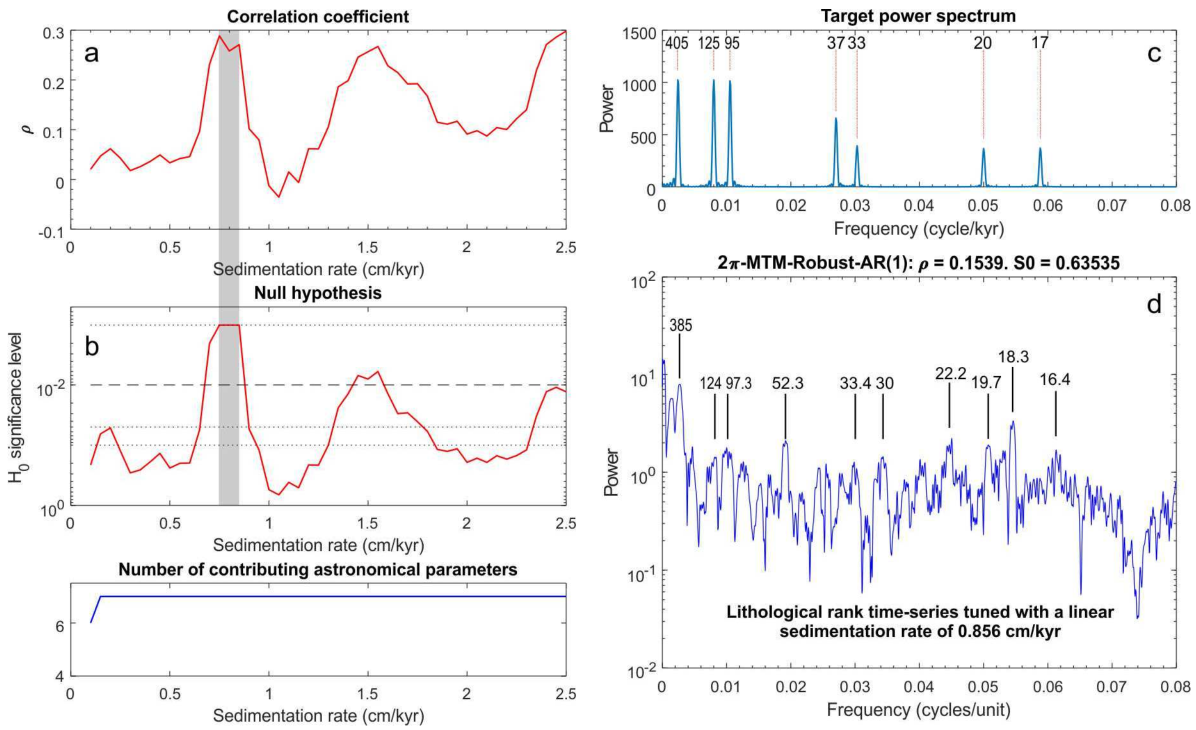

Supplementary Fig. 12: Results of a COCO procedure[2] on the Steinsodden time-series. a Correlation coefficient of frequency peaks identified in the original data in the depth domain (see Fig. 4B of the main text for the full spectrum) to orbital targets. **b** Likelihood of the null hypothesis “no Milankovitch influence on the dataset” given as significance levels. **c** Spectrum of the chosen orbital targets compared to **d** spectrum of our time-series tuned in time with a stationary sedimentation rate of 0.856 cm/kyr.

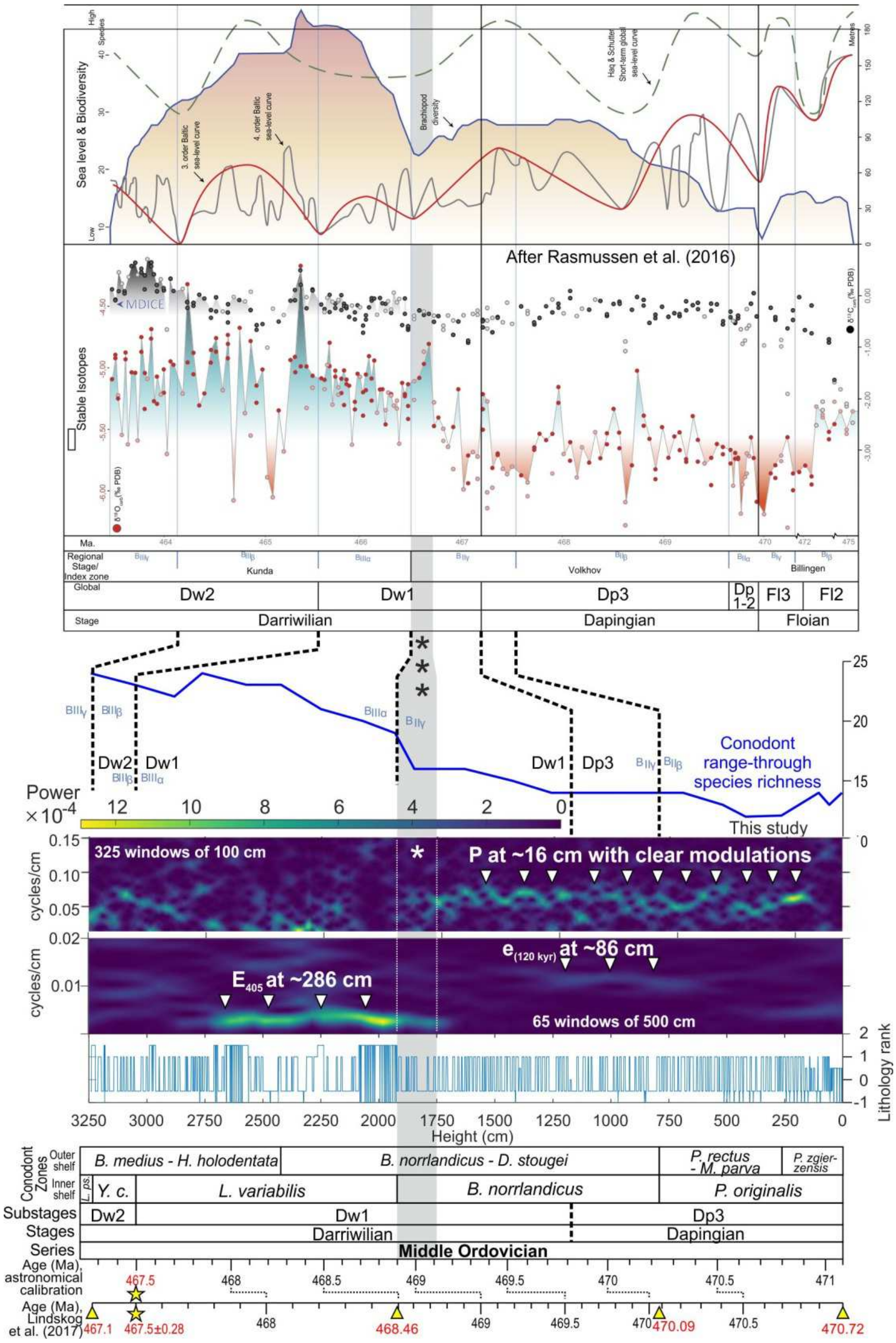

Supplementary Fig. 13: Correlation of our Steinsodden section to the isotopic results and synthetic scheme of Rasmussen et al.[3] We use the stratigraphy of our section to correlate (i) the timing of the shift from precession to 405 kyr as expressed in the evolutive spectrograms (this timing is highlighted by the white and black stars) and (ii) our conodont species richness, to (iii) the timing of the main oxygen isotope shift in brachiopod data and (iv) sea-level change and brachiopod diversity. Note the coincidence of the onset of a Middle Ordovician ice age (as shown by the

pronounced shift in brachiopod $\delta^{18}O$) and of the energy transfer from precession-dominated to a 405 kyr-dominated sedimentation.

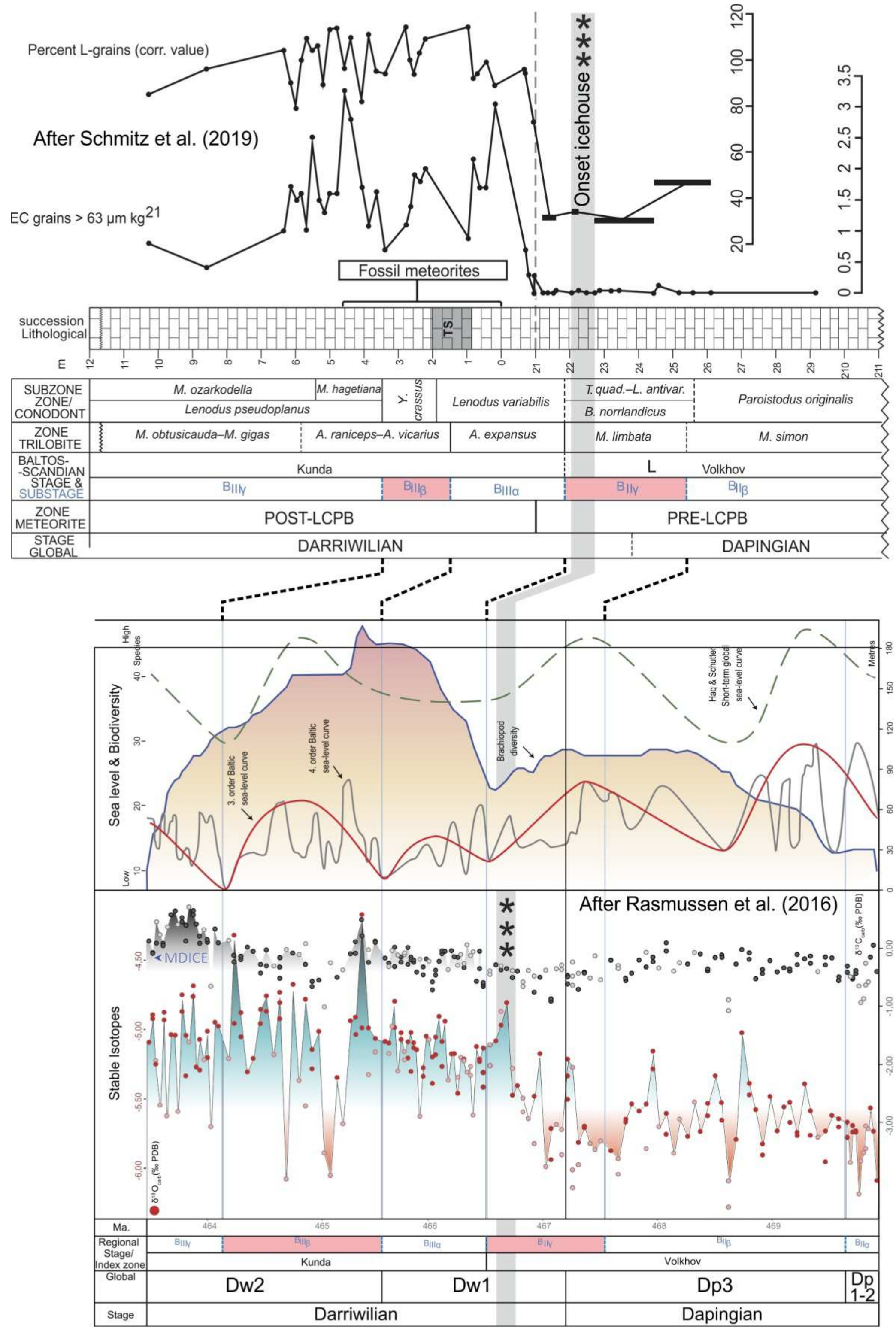

Supplementary Fig. 14: Correlation of Brachiopod diversity, stable isotopic results on Brachiopods (carbon and oxygen) and sea-level change[3] to the stratigraphical distribution of fossil meteorite

grains[4]. Our correlation supported by the similarly applied Baltoscandian regional stage and substages show that the onset of a Middle Ordovician icehouse precedes the onset of the LCPB.

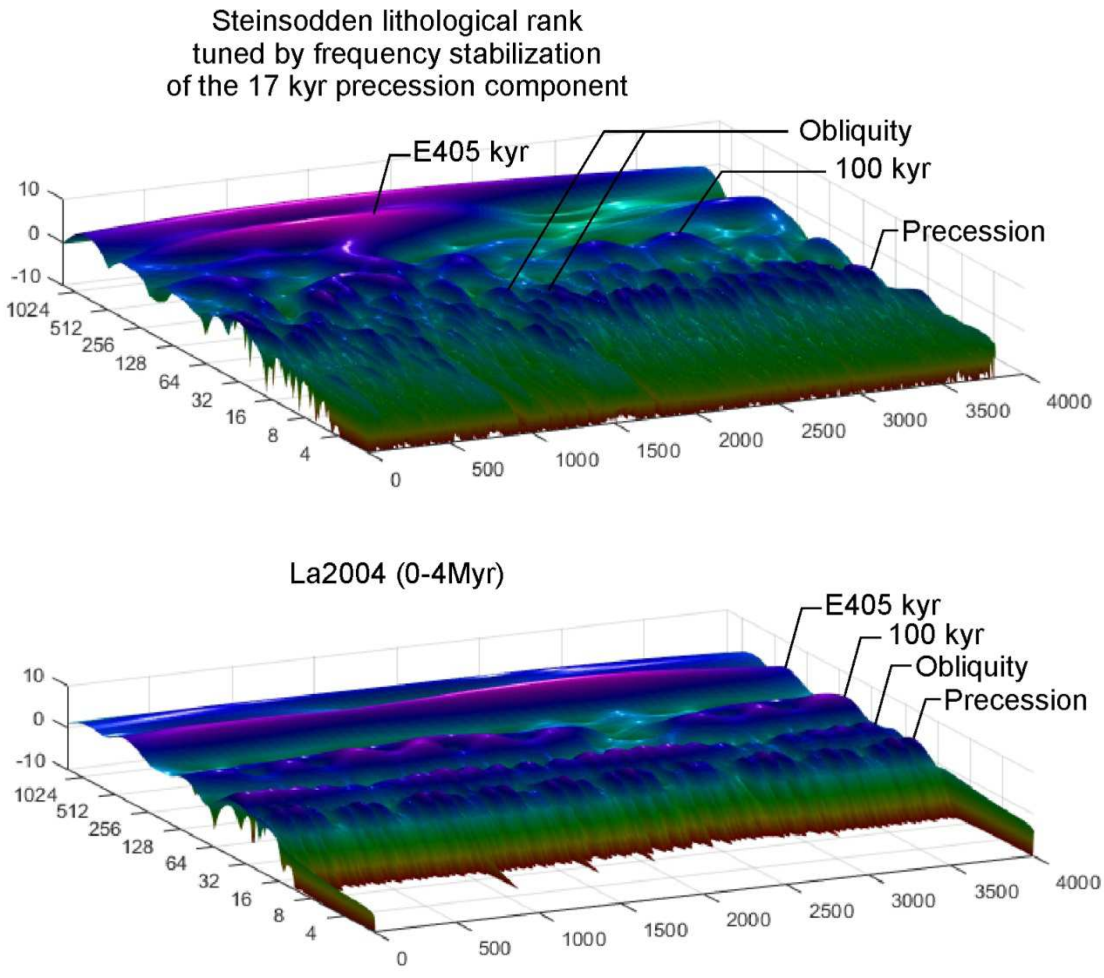

Supplementary Fig. 15: 3D wavelet transform views of our Middle Ordovician dataset compared to La2004 ETP model for the past 4 million years. This view shows the expression of changes in amplitude of the different components. Note the modulations in amplitude of the precession and 100 kyr components in both datasets. We used the script of Liebrand et al.[5] for wavelet analysis with modifications for 3D views as introduced by Li et al.[2].

References

1 Rasmussen, J. A. Conodont Biostratigraphy and Taxonomy of the Ordovician Shelf Margin Deposits in the Scandinavian Caledonides. *Fossils and Strata* **48**, 1–180 (2001).

2 Li, M., Kump, L. R., Hinnov, L. A. & Mann, M. E. Tracking variable sedimentation rates and astronomical forcing in Phanerozoic paleoclimate proxy series with evolutionary correlation coefficients and hypothesis testing. *Earth and Planetary Science Letters* **501**, 165–179 (2018).

3 Rasmussen, C. M. Ø. *et al.* Onset of main Phanerozoic marine radiation sparked by emerging Mid Ordovician icehouse. *Scientific Reports* **6**, 18884, doi:DOI: 10.1038/srep18884 (2016).

4 Schmitz, B. *et al.* An extraterrestrial trigger for the mid-Ordovician ice age: Dust from the breakup of the L-chondrite parent body. *Science Advances* **5** (2019).

5 Liebrand, D. *et al.* Cyclostratigraphy and eccentricity tuning of the early Oligocene through early Miocene (30.1–17.1 Ma): Cibicides mundulus stable oxygen and carbon isotope records from Walvis Ridge Site 1264. *Earth and Planetary Science Letters* **450**, 392–405 (2016).
